# Risk factors for non-adherent retained placenta after vaginal delivery: a systematic review

**DOI:** 10.1186/s12884-021-03721-9

**Published:** 2021-03-31

**Authors:** Alessandro Favilli, Valentina Tosto, Margherita Ceccobelli, Fabio Parazzini, Massimo Franchi, Vittorio Bini, Sandro Gerli

**Affiliations:** 1 Department of Obstetrics and Gynecology, AOUI Verona, University of Verona, Piazzale A. Stefani 1, 37126 Verona, Italy; 2 Department of Medicine and Surgery, Obstetrics and Gynecology, Centre of Perinatal and Reproductive Medicine, Santa Maria della Misericordia Hospital, University of Perugia, 06156 Perugia, Italy; 3 Department of Clinic and Community Science, Mangiagalli Hospital, University of Milan, 20122 Milan, Italy; 4 Department of Medicine and Surgery, Santa Maria della Misericordia Hospital, University of Perugia, 06156 Perugia, Italy

**Keywords:** Retained placenta, Morbidly adherent placenta, Vaginal delivery, Prolonged third stage of labor, Risk factors, Post-partum hemorrhage

## Abstract

**Background:**

Retained placenta represents a cause of maternal morbidity and mortality affecting 0.5–3% of all vaginal deliveries. The unpredictability of this condition makes difficult to develop predictive and preventive strategies to apply in clinical practice. This analysis collected and analyzed all known risk factors related to this obstetric complication.

**Methods:**

A systematic literature review for all original research articles published between 1990 and 2020 was performed. Observational studies about retained placenta risk factors published in English language were considered eligible. Conference abstracts, untraceable articles and studies focused on morbidly adherent placenta were excluded. The included articles were screened to identify study design, number of enrolled patients and retained placenta risk factors investigated. All stages of the revision followed the Preferred Reporting Items for Systematic Reviews and Meta-Analyses (PRISMA) Statement.

**Results:**

Thirty-five studies met the inclusion criteria. The reported retained placenta prevalence ranged from 0.5 to 4.8%. Maternal age, previous cesarean sections, previous dilation and curettage, previous retained placenta, labor induction, resulted as the most recurrent, independent risk factors for retained placenta. Previous estro-progestins therapy, morphological placental features (weight, shape, insertion of umbilical cord, implantation site), endometriosis, Assisted Reproductive Technologies, Apgar score are fascinating new proposal risk factors.

**Conclusions:**

Old and new data are not enough robust to draw firm conclusions. Prospective and well-designed studies, based on a well agreed internationally retained placenta definition, are needed in order to clarify this potential dramatic and life-threatening condition.

**Supplementary Information:**

The online version contains supplementary material available at 10.1186/s12884-021-03721-9.

## Background

Retained placenta (RP) affects 0.5–3% of all vaginal deliveries and is considered one of the major causes of primary and secondary postpartum hemorrhage (PPH), increasing maternal morbidity and mortality risk [1–5]. Incidence of RP varies greatly around the world depending on the population studied [5, 6]. Not only RP per se, but also its removal is associated with several complications such as infections, trauma of the urogenital tract, prolonged hospitalization and anesthesiological risks [7, 8].

The pathophysiology of this condition is not perfectly known and it is probably complex. Three major mechanisms for RP have been suggested: invasive placentation, placental hypoperfusion and inadequate myometrial contractility. The first one usually results from previous uterine trauma; the second mechanism could be related to incomplete spiral artery remodeling and shallow placentation. Lastly, localized retroplacental contractility failure was suggested to play a role in retention [2, 5, 9, 10].

At present, only a few number of risk factors for RP have been recognized with certainty. A previous retained placenta, older maternal age and prolonged use of oxytocin have been demonstrated to be correlated to RP. But instead, conflicting results have been reported about the role of parity, smoking, gestational diabetes as well as Assisted Reproductive Technologies (ART) [2, 3, 6, 7, 9]. This makes difficult to develop useful, concrete, predictive and preventive strategies.

The aim of this paper was to systematically search the relevant databased and give a description of all risk factors associated with retained placenta after vaginal delivery.

## Methods

We performed a systematic literature search for all original research articles using the following database: PubMed/MEDLINE, Embase, Scopus and Google Scholar. We used both free text and a MeSH search for keywords including “retained placenta”, “morbidly adherent placenta”, “vaginal delivery”, “prolonged third stage of labor”, “risk factors”, “post-partum hemorrhage”, published between 1990 and 2020. Observational studies published in English language (case-controls, cohorts, cross sectionals studies) were considered eligible if dealing with RP risk factors. Studies including placenta accreta were excluded, as it is a well-known RP cause, but our aim was to investigate risk factors behind a non-pathologically adherent placenta. As well, studies focused on trapped placenta were excluded. Trapped placenta is the condition in which the placenta is trapped behind a closing cervix; it is rare and more relevant in very preterm labor, and little is known about it [9]. Conference abstracts were not included in the analysis. If any data was missing, the investigator of the study was contacted by e-mail for additional information.

A PICOS (Patient, Intervention, Comparator, Outcome, Study) design structure was used to develop the study question and the inclusion/exclusion criteria. The question was: “What are the risk factors for retained placenta?”

All the design, analysis, interpretation of data, drafting and revisions followed the Preferred Reporting Items for Systematic Reviews and Meta-Analyses (PRISMA) Statement [11], available through the Enhancing the QUAlity and Transparency Of Health Research (EQUATOR) network. The review was registered in PROSPERO database (registration number: CRD42018102190).

Titles and/or abstracts of retrieved articles were screened to identify studies that potentially met the inclusion criteria. Duplicates were removed and the remaining references were examined independently by two authors (M. C. and V. T.). The full texts of these studies were retrieved and independently assessed for eligibility by other authors (A. F., F. P., M.F., V.B. and S. G.). Any disagreement between them over the eligibility of particular studies was resolved through collective discussion. A standardized form was used to extract data from the included studies for assessment of study quality and evidence synthesis. We selected information about study design, number of patients enrolled and risk factors for RP investigated.

## Results

The flow chart for search and identification of studies in the review is reported in Additional file 1 (PRISMA flow diagram). Using the reported search modality, 240 references were found. Duplicate references were 76 and therefore removed. A total of 164 studies were selected, but 4 were excluded due to the fact that insufficient data were available or were not provided by the authors. One hundred and fifty-eight papers were screened; 123 were excluded because considered outside the purpose. Lastly, 37 studies met our inclusion criteria and were eligible for the review. The design of the selected studies was as follows: 32 retrospective analysis, 4 prospective analysis and 1 study was both retrospective and prospective. The analyzed studies are summarized in Table 1, where different definitions of retained placenta are summarized.

**Table 1:** Relevant data of retrieved studies

Authors, year, Country	Study Design	Study period (years)	Overall deliveries (n)	RP diagnosis (min) and definition	Prevalence of RP	Management
Meyer, 2020 Israel, [12]	Retrospective	8	16,867	60, D	6.5% (including complete and partial retained placenta)	Active
Granfors, 2020 Sweden, [13]	Retrospective	6	49,598	NS, E	2.0%	NS
Rottenstreich, 2019 Israel [14]	Retrospective	13	170,009	40, C	2.70%	Active
Ruiter, 2019 Netherlands [15]	Retrospective	11	359,737	NS, E	2.7%, (first pregnancy), 17% (recurrence in second pregnancy)	NS
Favilli, 2018 Italy [16]	Retrospective	10	22,749	30, B	0.6%	Active
Rotem, 2018 Israel [17]	Retrospective	25	263,053	30, B	0.7% (Hypertenstìyve disorders) 0.6% (No hypertensyve disorders)	Active
Vannuccini, 2018 Italy [18]	Retrospective	5	1168 (Spontaneous pregnancies) 188 (ART pregnancies)	NS, E	6.30% (ART pregnancies) 2.8% (spontaneous pregnancies)	NS
Endler, 2017 Sweden [19]	Retrospective	40	494,000	30, B	2.4%, (index births) 3.2% (second generation births)	Observational + active
Greenbaum, 2017 Israel [9]	Retrospective	25	205,522	30, B	4.8%	Observational
Anteby, 2017 Israel [7]	Prospective	2	19,359	60, D	1.5%	Active
Alufi, 2017 Israel [6]	Retrospective	4	18,146	30, B	0.5%	Observational
Berlac, 2017 Denamark [20]	Retrospective	14	11,739 (women with endometriosis disease)	NS, E	2.20%	Observational
Aziz, 2016 New Jersey [21]	Retrospective	5	769	NS, E	1.05% (ART pregnancies) 1% (spontaneous pregnancies)	NS
Sarit, 2016 Israel [22]	Retrospective	0,5	4227	60, D	1.9%	Active
Jackson, 2015 California [23]	Retrospective	11	193 (spontaneous pregnancies 185 (ART pregnancies)	NS, E	0% (spontaneous pregnancies) 2.7% (ART pregnancies)	Observational
Shinar, 2015 Israel [24]	Retrospective	5	25,160	60, B	9.00%	Active
John, 2015 Nigeria [25]	Retrospective	5	15,789	30, B	0.59%	Observational
Coviello, 2015 USA [3]	Retrospective	6	228,562	30, B	1.12%	Observational
Torricelli, 2015 Italy [26]	Prospective	3	2354	NS, E	NS	Observational
Ashwal, 2014 Israel [27]	Retrospective	5	33,925	30, B	1.4%	Active
Endler, 2014 Sweden [2]	Retrospective	12	386,607	NS, E	2.17%	Active
Magann, 2013 USA [28]	Retrospective	2	452	15, A	NS	Observational
Nikolajsen, 2013 Denmark [29]	Retrospective	9	10,334	30, B	2.60%	Observational
Endler, 2012 Sweden [30]	Retrospective	3	16,209	30, B	2.6%	Active
Farhi, 2010 Israel [31]	Retrospective	2	280	NS, E	NS	NS
Obajimi, 2009 Nigeria [32]	Retrospective	5	4980	30, B	2.13%	Observational
Rizwan, 2009 Pakistan [33]	Retrospective + prospective	3	8782	30, B	1.02%	Active
Owolabi, 2008 Nigeria [34]	Prospective	7	6160	30, B	1.9%	Observational + active
Panpaprai, 2007 Thailand [35]	Retrospective	3	234 (78 cases, 156 controls)	30, B	NS	Observational
Chhabra, 2002 India [8]	Retrospective	15	29,784	NS, E	0.23%	Observational + active
Titz, 2001 Australia [36]	Retrospective	7	3734	60, D	3.00%	Observational
Adelusi, 1997 Saudi Arabia [37]	Retrospective	5	22,045	NS, E	0.06%	Observational
Soltan, 1997 Saudi Arabia [38]	Retrospective	6	26,315	60, D	0.55%	Observational
Golan, 1996 Israel [39]	Retrospective	NS	NS	NS	NS	NS
Dombrowski, 1995 Michigan [40]	Prospective	9	45,852	30, B	2.00%	Active
Combs, 1991 California [41]	Retrospective	11	12,979	30, B	3.3%	Observational
Romero,1990 Connecticut [42]	Retrospective	2	792	30, B	3.40%	Observational

*NS* Not Specified, *RP* Retained Placenta, *ART* Assisted Reproductive Technologies

RP Definition A: third stage of labor lasting > 15 min

RP Definition B: failure to deliver placenta after a 30-min duration of an uncomplicated third stage

RP Definition C: failure to deliver placenta after a 40-min or more duration of an uncomplicated third stage

RP Definition D: failure to deliver placenta after a 60-min or more duration of an uncomplicated third stage

RP Definition E: placenta that requires manual removal

### Prevalence

As reported in Table 1, the RP prevalence ranged from 0.5 to 4.8%. Nevertheless, Shinar et al. described a RP prevalence of 9%. This gap could be explained by the inclusion of placental manual extractions due to vaginal bleeding or incomplete placental separation, which have been performed before the one-hour waiting, expected by the Centre policy for the active management of the third stage of delivery.

### Demographic risk factors

Parity, advanced maternal age, and ethnicity have been considered well-established RP risk factors. Coviello et al. observed a statistically significant association with multiparity (OR 4.56, 95% CI 2.08–9.94) [3]. However, Ashwal observed that women with RP had lower parity (OR 0.79, 95% CI 0.68–0.91) [27]; Endler that parity of two or more had a protective effect (OR 0.40, 95% CI 0.24–0.70) [30] and Combs reported a higher risk for RP in nulliparous versus multiparous (*p* < 0.01) and in para ≥5 vs para < 5 (*p* < 0.05) [41].

Frequency of RP was higher (37.7%) in women aged 26–30 than in patients aged 34–40 (13.3%) [33]. According to Coviello, RP was associated with older maternal age (27.5 years versus 26.6 years, *p* < 0.001) [3].

A higher rate of Jewish ethnicity (OR 1.12, 95% CI 1.07–1.17) in the RP group was also found [9]. Otherwise, RP was related with non-Hispanic black or Asian women [3].

Compared with women with spontaneous placental expulsion, those with RP were with a higher Body Mass Index (BMI) [2, 9]. Endler showed that 20.2% of women with RP were overweight (OR 1.13, 95% CI 1.07–1.21) and 7.9% of women were obese (OR 1.28, 95% CI 1.16–1.40) [2].

### Smoking

Smoking during pregnancy was not significantly related to RP risk. Endler showed that 92.6% of women with RP did not smoke, while 7.6% were smokers [2]. Likewise, Greenbaum reported that women with RP were smokers in 0.5%, while women with normal placental delivery had a smokers rate of 1.0% (OR 0.51,95% CI 0.39–0.67) [9]. Even, Endler observed that smoking could have a protective effect on the prevalence of RP, speculating an effect of reduction of placental apoptotic process, similar to that of carbon monoxide in preventing preeclampsia [30].

### Previous uterine surgery

A history of a previous uterine surgery, like cesarean section (CS), dilation and curettage (D&C) and myomectomy, has been highlighted as independent RP risk factor (OR 8.82, 95%CI 8.35–9.31 for previous CS; OR 12.80, 95%CI 10.57–15.50 for previous D&C; OR 1.97, 95%CI 1.05–3.71 for history of other instrumental procedures) [9].

### Recurrence of RP

Authors reported the history of RP as a risk factor for recurrence in subsequent pregnancies [6, 28, 32, 33]. Nikolajsen et al. observed an increased risk among women with a history of RP after vaginal delivery with a prevalence ranging from 2.8 to 7.0% [29]. Endler found that there is an intergenerational RP recurrence, on the maternal and paternal side [19]. In this study, mothers born in a pregnancy complicated by RP had an increased risk of RP when giving birth themselves (OR 1.66, 95% CI 1.52–1.82). Fathers born in a pregnancy complicated by RP had an increased risk of fathering a pregnancy complicated by RP (OR 1.23, 95% CI 1.07–1.41).

Rottenstreich described the higher risk of RP recurrence in multiple consecutive deliveries. RP was diagnosed in 2.7% deliveries and 13.8% women had at least one RP recurrence in any of their subsequent deliveries: 85.7% had one additional episode, 11.3% two more events, 2.3% three more events [14]. Ruiter found that among women with a first pregnancy complicated by manual removal of placenta, 17% underwent the same procedure in the second pregnancy (OR 6.1, 95% CI 5.6–6.7) [15].

### Uterine pathologies

Women with history of endometriosis showed a higher RP risk (OR 3.1, 95% CI 1.4–6.6) [20]. Moreover, authors described an association between morphological uterine anomalies and RP [5, 32]. At this regard, Golan found incomplete uterine septum at hysteroscopic examination in about 15% of women who underwent a manual removal of the placenta [39]. Uterine fibroids could be another RP risk factor: Rizwan showed that 7.7% patients with RP had uterine fibroids [33].

### Obstetrical risk factors

Concerning labor management, oxytocin use and its exposure time during labor was related with RP. A retrospective study demonstrated that an increasing duration of oxytocin administration resulted as a RP risk factor; nevertheless, this was not confirmed by the logistic regression analysis [16]. This data is in contrast with previous results [30], asserting that it was not oxytocin per se to be involved in RP, but the consequences of its prolonged use that could exhaust the contractility myometrial force. Oxytocin use for 195–415 min led to a risk of RP two times higher than labor without it (OR 2.00, 95% CI 1.20–3.34), whereas its use for more than 415 min related to a risk more than six times higher (OR 6.55, 95% CI 3.42–12.54). Epidural analgesia, also, appeared to be significantly associated with placental disorders when added to oxytocin.

Instrumental vaginal delivery is another potential RP risk factor reported in several studies (OR 1.54, 95% CI 1.45–1.63), (OR 1.89, 95% CI 1.48–2.41), (OR 1.54, 95% CI 1.47–1.62), (*p* < 0.0001) [2, 16, 19, 27].

Pregnancies conceived with Assisted Reproductive Technologies have a significantly higher rate of placentation defects (OR 2.63, 95% CI 1.31–5.26) [18]. Jackson showed a higher risk for RP in ART pregnancies than spontaneous conceptions (2.7% vs 0%, *p* = 0.02) and a higher risk for ART gestations conceived through donor oocytes versus autologous oocytes (3.3% vs 1.6%) [23]. However, Aziz showed an increased frequency of manual placental extraction only within women who underwent embryo transfer with no exposure to controlled ovarian hyperstimulation [21].

The Great Obstetrical Syndromes (GOS), a collective name for several pregnancy complications including gestational diabetes, hypertensive disorders, pre-eclampsia, intrauterine growth restriction (IUGR), preterm delivery and stillbirth, could be related to RP [2, 27]. RP was found strongly associated with preterm labor, particularly less than 27 weeks of gestation with a relative risk ranging from 6 to 13 [41, 42]. Obajimi observed that about 56.7% RP cases had a lower mean gestational age at delivery (34.29 +/− 6.02 weeks) than RP cases at term (43.3%) [32]. Dombrowski found the frequency of RP markedly increased among 20 to 26 weeks gestation (25.4%, OR 20.8, 95% CI 17.1–25.4) and among gestations < 37 weeks (4.7%, OR 3.8, 95% CI 2.6–3.5) compared with term gestations (1.6%) [40]. Romero et al. found that the incidence of RP was significantly higher in women with preterm vaginal delivery than in women with term vaginal delivery (9.1% against 1.1%, OR = 9.25, *p* < 0.0001) [42].

Concerning fetal characteristics, RP risk was increased by a lower birth weight, fetal growth restriction and stillbirth, which reflects the possible defective placentation on the fetal well-being [2, 3].

The strength of the association between diabetes (pre-gestational and gestational) and RP is difficult to evaluate [2]. Nevertheless, authors identified GDM as a risk factor for RP (OR 1.31, 95% CI 1.21–1-43, *p* < 0.001). Greenbaum considered GDM among the variables related to the placental hypoperfusion. This mechanism has been suggested as a common insult of both complications during pregnancy and abnormal detachment of the placenta during childbirth [9].

About hypertensive disorders, Rotem described a higher rate of third stage complications among hypertensive parturients (4.7% versus 4.0%, *p* < 0.001) and an independent correlation between RP and preeclampsia (OR 1.34, 95% CI 1.10–1.63) [17]*.*

### Placental histological data

Endometritis and chorioamnionitis are associated with RP as possible causes of inadequate uterine contractility (*p* < 0.001) [9].

Several studies demonstrated a relation between RP and placental weight less than 500 g [20, 35]. The analysis of placental morphological characteristics after discharge allowed to confirm a statistically significant association between RP and lower placental weight (*p* = 0.0001) [16].

### “New proposed” RP risk factors

A prospective study demonstrated that placental location, determined by ultrasonographic examination, influenced the onset and progress of labor and postpartum outcome: women with anterior placenta showed a prolonged third stage of labor (*p* = 0.01), a higher risk of manual placental removal (*p* = 0.003) and a higher rate of PPH in vaginal deliveries (*p* = 0.02) [26]. Otherwise, an association of RP and placental implantation site was demonstrated (*p* = 0.018); a higher rate of RP was shown when the placental site was anterior (anterior 47.9%, fundic 22.7%, posterior 20% and lateral 9.2%) [16]. Meyer et al. found that lateral and fundal location of placenta were both associated with increased risk of third stage placental complications (partial or complete retained placenta (OR 2.05, 95% CI 1.35–3.11; OR 1.65, 95% CI 1.01–2.26, respectively) [12]. Finally, Granfors conducted an analysis, including women with a previous cesarean section, demonstrating that the risk for retained placenta was not increased with anterior compared with non-anterior placental location (OR 0.84, 95% CI 0.60–1.20) [13].

New data derived from umbilical cord insertion. A more frequent central umbilical cord insertion (45.2%) in the RP group, rather than the paracentral (21.5%), velamentosa (9.6%) and marginal (15.6%) insertions was observed (*p* = 0.0001) [16].

Notably, it was found that a lower Apgar score at 1 min (*p* = 0.0001) and at 5 min (*p* = 0.02) were strongly related with RP [16].

As a novelty, a previous estroprogestin therapy resulted to be a protective factor for retained placenta (OR 0.58; 95% CI 0.31–1.09, *p* = 0.09) [16].

## Discussion

### Main findings

Retained placenta risk factors which have been assessed are advanced maternal age, previous RP, previous surgical uterine procedures and labor induction with oxytocin. Previous estro-progestins therapy, morphological placental features, endometriosis, ART, Apgar score are new proposal risk factors awaiting further confirmation. Table 2 shows the detailed analysis between every risk factor and the included studies.

**Table 2:** RP risk factors analysis

Authors	ART	Hypertensive disorders	GDM	Preterm birth	Stillbirth	Infections	Labour Induction	PROM	Epidural Analg	IVD	Placental data	E/P	Maternal age	Parity	Previous CS	Previous D&C	Previous RP
**Meyer, 2020,** **[**12**]**	1.45 (1.25–1.69)	4.00 (2.29–6.99)	NS	NS	NA	NA	1.32 (1.14–1.52)	NA	1.31 (1.12–1.54)	NA	NA	NA	*P* < 0.001	–	NA	NA	NA
**Granfors, 2020,[**13**]**	1.46 (0.63–3.37)	NA	NA	NA	NA	NA	NA	NA	NA	NA	0.84(.06–1.2)	NA	1.54(0.9–2.63)	–	–	NA	NA
**Rottenstreich, 2019** **[**14**]**	NA	NS	0.4 (0.07–1.3)	NS	NS	NS	1.61 (1.16–2.24)	NS	1.45 (1.10–1.90)	NA	NA	NA	NS	NS	NS	NA	4.9 (3.90–6.32)
**Ruiter, 2019** **[**15**]**	NA	*P* < 0.001	NA	*P* < 0.001	NA	NA	NA	NA	NA	NA	NA	NA	*P* < 0.001	NA	NA	NA	6.1 (5.6–6.7)
**Favilli, 2018** **[**16**]**	NS	NS	NS	NA	NA	NA	4.29 (1.83–10.02)	NS	NA	*P* = 0,00001	1.33 (0.96-1.84)	0.58(0.31-1.09)	1.04 (0.98-1.11)	NA	NS	1.92 (1.04-3.54)	NA
**Rotem, 2018** **[**17**]**	NA	1.34 (1.10–1.63)	NA	NA	NA	NA	NA	NA	NA	NA	NA	NA	NA	NA	NA	NA	NA
**Vannuccini, 2018** **[**18**]**	2.63 (1.31–5.26)	NA	NA	NA	NA	NA	NA	NA	NA	NA	NA	NA	NA	NA	NA	NA	NA
**Enlder, 2017** **[**19**]**	NA	1.78 (1.64–1.93)	NA	5.03 (4.01–6.31)	2.71 (2.26–3.25)	NA	NA	2.01 (1.82–2.22)	NA	1.54 (1.47–1.62)	NA	NA	1.90 (1.77–2.03)	0.80 (0.77–0.82)	NA	1.61 (1.41–1.85)	1.66 (1.52–1.82)
**Greenbaum, 2017** **[**9**]**	NS	1.25 (1.14-1.38)	1.31 (1.21-1.43)	NA	2.34 (1.98-2.77)	3.35 (2.78-4.04)	NA	NA	NA	NA	NA	NA	NA OR	NA OR	8.82 (8.35-9.31)	12.8 (10.75–15.5)	66.47(63.09–70.04)
**Anteby, 2017** **[**7**]**	NA	NS	NA	NA	NA	NA	1.80 (1.12-2.88)	NA	3.49 (2.14-5.70)	NA	NA	NA	1.08 (1.03-1.12)	P 0,02 *P* < 0,001	NA	1.24 (0.84-1.83)	9.27(3.15-27.31)
**Alufi, 2017** **[**6**]**	NA	NA	NA	NA	NA	NA	NA OR	NA	NS	NA	NA	NA	NA OR	NA OR	NA	NA	9.8 (1.2-80.6)
**Berlac, 2017** **[**20**]**	NA	NA	NA	NA	NA	NA	NA	NA	NA	NA	NA	NA	NA	NA	NA	NA OR	NA
**Aziz, 2016** **[**21**]**	5.6 (2.2–13.8)	NA	NA	NA	NA	NA	NA	NA	NA	NA	NA	NA	NA	NA	NA	NA	NA
**Sarit, 2016** **[**25**]**	NA	NA	NA	NA	NA	NA	NA	NA	NA	NA	NA	NA	1.07 (1.03-1.10)	NA	1.97 (1.03- 3.78)	NA OR	NA OR
**Jackson, 2015** **[**23**]**	NA OR	NA	NA	NA	NA	NA	NA	NA	NA	NA	NA	NA	NA OR	NA	NA	NA	NA
**John, 2015** **[**25**]**	NA	NA	NA	NA OR	NA	NA	NA	NA	NA	NA	NA	NA	NS	NS	NA	NA OR	NA OR
**Coviello, 2015** **[**3**]**	NA	NA	NA	NA OR	5.6 (3.10- 10.37)	*P* < 0.001	NS	NA	NA OR	NA	NA	NA	*P* < 0,001	4.56 (2.08- 9.94)	NA OR	NA	NA
**Torricelli, 2015** **[**26**]**	NA	NA	NA	NA	NA	NA	NA	NA	NA	NA	*P* = 0,003	NA	NA	NA	NA	NA	NA
**Shinar, 2015** **[**12**]**	NA	NA	NA	*P* < 0.0001	NS	1.73 (1.2–2.4)	NS	NS	NS	NA	NA	NA	*P* = 0.017	1	NS	1.25(1.05–1.47)	NA
**Ashwal, 2014** **[**27**]**	1.29 (0.86–1.92)	1.72 (1.00- 2.83)	NS	NS	NA	NA OR	1.84 (1.30- 2.59)	1.25 (0.96–1.63)	1.60 (1.27- 2.00)	1.89(1.48- 2.41)	NA	NA	1.05(1.03–1.07)	0.79 (0.68- 0.91)	1.71 (1.23- 2.36)	1.22(1.03–1.49)	NA
**Endler, 2014** **[**2**]**	1.80 (1.60- 2.04)	1.72 (1.33- 2.22)	1.13 (0.88- 1.45)	2.35 (1.97- 2.81)	1.71 (1.28- 2.29)	NA	1.63 (1.52- 1.74)	1.44 (1.33- 1.55)	1.21 (1.15- 1.27)	1.54(1.45- 1.63)	NA	NA	2.52 (2.33- 2.72)	NS	NA	NA	NA
**Magann, 2013** **[**28**]**	NA	NA	NA	NS	NA	NA OR	NS	NA	NA	NA	NA	NA	NS	NA OR	NS	NS	NA OR
**Nikolajsen, 2013** **[**29**]**	1.11 (0.05- 8.8)	NS	NS	1.04 (0.13- 21.88)	NA	NA	1.93 (0.72–4.80)	NA	NA	NA	NA	NA	2.02 (0.84- 4.64)	NS	NA	NA	5.49 (2.57- 12.42)
**Endler, 2012** **[**30**]**	NA	2.85 (1.20- 6.78)	5.05 (0.59- 43.41)	3.28 (1.60- 6.70)	NA	NA OR	6.55 (3.42- 12.54)	NA	0.68 (0.47- 0.98)	1.18(0.75-1.84)	NA OR	NA	NS	NS	NA OR	2.62 (1.31- 5.20)	12.61 (3.61- 44.08)
**Farhi, 2010** **[**31**]**	NA OR	NA	NA	NA	NA	NA	NA	NA	NA	NA	NA	NA	NA OR	NA	NA	NA	NA
**Obajimi, 2009** **[**32**]**	NA	NA	NA	NA OR	NA	NA	NA	NA	NA	NA	NA	NA	NS	NS	NA OR	NA OR	NA OR
**Rizwan, 2009** **[**33**]**	NA	NS	NA	NA	NA	NA	NA OR	NA	NA	NA	NA	NA	NA OR	NA OR	NS	NS	NA OR
**Owolabi, 2008** **[**34**]**	NA	NA	NA	3.12 (1.12- 8.68)	NA	NA	NA	NA	NA	NA	2.91 (1.34- 6.32)	NA	7.10 (1.5- 32.40)	6.63 (1.88- 23.40)	12.00 (2.05- 70.19)	4.44 (1.69- 11.63)	15.22 (3.30- 70.19)
**Panpaprai, 2007** **[**35**]**	NA	NA	NA	NS	NA	NA	NS	2.2 (1.2- 4.1)	NS	NA	NA	NA	1.06 (1.01- 1.11)	NS	NA	2.95 (1.7- 9.9)	NS
**Chhabra, 2002** **[**8**]**	NA	NA	NA	NA OR	NA	NA	NS	NA	NS	NS	NA	NA	NA OR	NA OR	NA OR	NA OR	NA OR
**Titz, 2001** **[**36**]**	NA	NA	NA	5.6 (1.1- 26.8)	NA	NA	NS	NA	NA	NA	NA	NA	NS	NS	NS	NS	9.8 (1.1- 85.5)
**Adelusi, 1997** **[**37**]**	NS	NA	NA	NA OR	NA	NA	NA OR	NA	NA	NA	NA OR	NA	NS	NA OR	NA OR	NA OR	NA OR
**Soltan, 1997** **[**38**]**	NA	NA	NA	13.95 (2.15–12.55)	NA	NA	3.32 (1.36- 8.20)	NA OR	NS	NA	3.42 (2.18- 5.36)	NA	4.77 (2.03- 11.37)	3.05 (1.43- 6.55)	5.52 (2.09- 15.07)	9.06 (4.39- 19.02)	28.98 (3.91- 123.09)
**Golan, 1996** **[**39**]**	NA	NA	NA	NA OR	NA	NA	NA	NA	NA	NA	NA	NA	NA	NA	NA	NA OR	NS
**Dombrowski,1994** **[**40**]**	NA	NA	NA	3.0 (2.6–3.5)	NA	NA	NA	NA	NA	NA	NA	NA	NA	NA	NA	NA	NA
**Combs, 1991** **[**41**]**	NA	NA OR	NA	NA OR	NA	NS	NA OR	NS	NS	NA	NA	NA	NS	NA OR	NA OR	NA OR	NA
**Romero, 1990** **[**42**]**	NA	NA	NA	NA OR	NA	NA	NA	NA	NA	NA	NA	NA	NA OR	NS	NA	NA	NA

Numerical data are reported as OR (95% CI)

*NA* Not Available data, *NS* Not significant data, *NA OR* Not Available Odds Ratio, *P p* value, *RP* Retained Placenta, *ART* Assisted Reproductive Technologies, *CS* Cesarean Section, *D&C* Dilation and Curettage, *PROM* Premature Rupture of Membranes, *GDM* Gestational Diabetes Mellitus, *IVD* Instrumental Vaginal Delivery, *E/P* Estro-progestin

### Strengths and limitations

The studies focused on RP risk factors are few and significantly differ in methodology design, inclusion and exclusion criteria and quality. Moreover, heterogeneity of RP definitions makes difficult to build an adequate dataset to meta-analyze.

This consideration supports the certainty that not similar definitions of RP are given in different settings [36]. Timing differences between countries were clearly shown in a European survey, comparing policies about manual removal of placenta at vaginal delivery: in the absence of bleeding the interval until manual removal of placenta varies from less than 30 min to more than 60 min [43].

Recently, the increased application of an active management of third stage of labor has brought to a significant shortening of the median duration of third stage of labor [43]. Shinar believed that a 30 min cut off is reasonable because he found 3% of deliveries with complication when third stage lengthened beyond 30 min [24].

Some studies do not report the percentage of retained placenta after a well-defined time period but manual removal rates [2, 8, 15, 31, 37].

The most widely shared definition of RP is the condition where the placenta is retained after 30 min of active management of third stage of labor or 60 min of physiological management [44].

### Interpretation

Although RP is a potentially life-threatening event, it is most likely under-reported. In developed countries it is more common (about 3% of all vaginal deliveries), but correlated with a lower mortality rate than in less developed ones (0.1% of deliveries with 10% of fatality rate) [1]. The reason of this trend seems to be the higher frequency of uterine surgeries, medicalization of labor and ART pregnancies in more developed countries [21, 30].

The great part of studies confirmed a well-established RP association between socio-demographic factors. The relation of an advanced maternal age and a higher number of pregnancies with RP could be explained by the substitution of myometrial fibers replaced by fibrous tissue, predisposing to uterine atony [37]. Women with increased BMI could be disposed to an increased oxidative stress, but whether this phenomenon could lead to changes in placental physiology is still unknown.

The association between placental retention and prior uterine surgery can be explained by the injuries of the endometrial-myometrial junction and the consequent anomalous implantation [34, 37, 38]. Instrumental procedures in non-pregnant uteri were associated with high risk of histologic evidence of abnormal placental implantation and invasiveness in subsequent pregnancies. These cases were three times more prevalent in placenta samples of women with RP than in those with normal placental expulsion (12.3% vs. 4.3%) [9]. Another theory, especially referred to previous CS, is a contractile failure in the retroplacental area as a result of uterine scar [34, 37].

Endometriosis has been found as a RP risk factor: functional and structural abnormalities in the inner myometrium have been demonstrated in Magnetic Resonance Imaging scans and in biopsy. This may lead to defective transformation of spiral arteries and abnormal placentation. Moreover, gynecological surgery for endometriosis seemed to add a further increased risk [20].

In case of uterine fibroids, the uterine cavity distortion or the anomalous placental adhesion at the level of the fibrous septum could explain a pathological placental detachment in the third stage of labor [33, 39].

Labor and delivery management are involved in other possible biological pathways of RP. Epidural analgesia, oxitocyn use, instrumental delivery can be related with uterine inadequate contractility [22, 30]. Oxytocin decreases glutathione and its prolonged use may lead to oxidative stress, predisposing a more vulnerable placenta to retention [30]. Epidural analgesia, seems to depress myometrial contraction, acting on the autonomic nervous system [22, 27, 38].

The association between operative delivery and RP, could be explained by the prolongation of the second stage of labor, caused in turn by a myometrial fatigue, but also by the occurrence of a dystocia complicating labor [16, 27].

Retained placenta may be more frequent in ART pregnancies because of the high estradiol level at the time of embryo-transfer. These patients were characterized by a high rate (20.8%) of pregnancy complications related to abnormal placentation [31]. The observed higher rate of placental defects could be also explained with advanced maternal age, ART itself and/or antigenic dissimilarity among oocyte donor and recipient [23].

The same abnormal placentation is usually considered in the Great Obstetrical Syndromes: imbalanced placental perfusion, oxidative stress and apoptosis in placental tissue [1, 2], and chronic inflammation status should be considered in hypertensive disorders and in GDM. Preterm birth and IUGR could be closely related to RP through an intrauterine inflammation in the placental site [42]. Besides to the vascular mechanisms described above, animal studies suggest a complex etiology linked to abnormal placental maturation, oxidative stress, imbalanced prostaglandin E2/F2 alpha ratio and decreased steroid hormone receptor status [2]. Indeed, there is an higher frequency of lower placental weight in case of RP, that could be explained by the presence of infarcts or fibrinoid degeneration of decidual arterioles [34, 37, 38]. This may derive as a result of the pathophysiologic origin of RP, thus as a defective placentation determining a regional incomplete vascular remodelling, excessive oxidative stress and emphasis of apoptotic process.

The relation between estroprogestin therapy and RP may be due to a positive effect on basal membrane induced by a stable concentration of hormones on the endometrium and myometrium contractility, or a reduction of incidence of uterine curettage, thanks to a reduced risk of unwanted pregnancy. Concerning the Apgar score, it was speculated that this evidence could be related to a fetal distress, determined by a second stage prolongation or by an abnormal placental implantation site [16]. This last hypothesis was in agreement with previous reports stating a condition of IUGR and/or lower mean birth-weight related to RP [3]. This data should be interpreted with caution because they could be potentially influenced by the gestational period at the delivery.

The characteristics of the “modern obstetric population” (advanced maternal age, nulliparity, higher medicalization of labor, ART pregnancies, endometriosis history) are interesting fields of investigation to try to fill the gaps on RP etiopathogenesis. In light of recent studies on endometriosis, ART procedures and use of estrogen-progestins therapy [16, 20, 23], we can hypothesize a relevant role of hormonal influence in the RP pathophysiology. Future investigations, particularly in the biomolecular area, would be useful to better understand, confirm or not the few available data.

## Conclusions

This review should remind and aware clinicians on the relevant RP risk factors, in order to improve preventive measures, management and reduce maternal morbidity and mortality. “Old and new data” are not enough robust to draw firm conclusions. Further studies are needed to provide more data on this issue so conduction of a meta-analysis would be possible.

## Supplementary Information

**Additional file 1: Figure 1.** Flow diagram of studies identified in the systematic review.

## Data Availability

All data generated or analyzed during this study are included in this published article and its supplementary information files.

## Abbreviations

RP: Retained Placenta
PPH: Post-partum hemorrhage
PRISMA: Preferred Reporting Items for Systematic Reviews and Meta-Analyses
EQUATOR: Enhancing the QUAlity and Transparency Of Health Research
BMI: Body Mass Index
CS: Cesarean section
D&C: Dilation and curettage
ART: (Assisted Reproductive Technologies
(GOS): Great Obstetrical Syndromes
GDM: Gestation Diabetes Mellitus
IUGR: Intra-Uterine Growth Restriction
