# Supplementary material for: Risk factors for non-adherent retained placenta after vaginal delivery: a systematic review
Source: BMC Pregnancy Childbirth. 2021 Mar 31;21:268. doi: 10.1186/s12884-021-03721-9 (PMC8015016; doi:10.1186/s12884-021-03721-9)
Supplement: Supplementary file 1 — Additional file 1: Figure 1. Flow diagram of studies identified in the systematic review. [file 12884_2021_3721_MOESM1_ESM.docx]

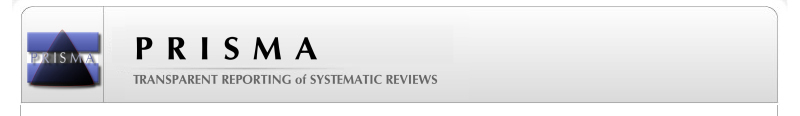
**PRISMA 2009 Flow Diagram**

Records identified through database searching
(n = 240)

Records screened
(n = 160 )

Full-text articles assessed for eligibility
(n = 37)

)

Studies included in qualitative synthesis
(n = 37 )

**Identification**

**Screening**

Additional records identified through other sources
(n = 0 )

Records after duplicates removed
(n = 164 )

Records excluded
(n = 4 )

Full-text articles excluded, with reasons: concerning placenta accreta spectrum, management/ diagnosis of retained placenta, retained placenta after first or second trimester abortion, relation retained placenta- postpartum hemorrhage
(n = 123 )

**Eligibility**

Figure 1. Flow diagram of studies identified in the systematic review.

*From:*  Moher D, Liberati A, Tetzlaff J, Altman DG, The PRISMA Group (2009).

*P*referred *R*eporting *I*tems for *S*ystematic Reviews and *M*eta-*A*nalyses: The PRISMA Statement. PLoS Med 6(6): e1000097. doi:10.1371/journal.pmed100009

**For more information, visit** [www.prisma-statement.org](http://www.prisma-statement.org/)
